# DBS in the restoration of motor functional recovery following spinal cord injury

**DOI:** 10.3389/fneur.2024.1442281

**Published:** 2024-12-04

**Authors:** Wen-yuan Li, Wen-rui Qu, Yi Li, Shu-ying Wang, Dong-ming Liu, Ling-xiao Deng, Ying Wang

**Affiliations:** ^1^Mudanjiang North Medicine Resource Development and Application Collaborative Innovation Center, Mudanjiang, China; ^2^Institute of Neural Tissue Engineering, Mudanjiang University of Medicine, Mudanjiang, China; ^3^Department of Hand Surgery, The Second Hospital of Jilin University, Changchun, China; ^4^Department of Neurology, Mudanjiang First People’s Hospital, Mudanjiang, China; ^5^Spinal Cord and Brain Injury Research Group, Stark Neurosciences Research Institute, Indiana University School of Medicine, Indianapolis, IN, United States

**Keywords:** deep brain stimulation, neural circuits, neuromodulation, neuroplasticity, spinal cord injury, motor function

## Abstract

The landscape of therapeutic deep brain stimulation (DBS) for locomotor function recovery is rapidly evolving. This review provides an overview of electrical neuromodulation effects on spinal cord injury (SCI), focusing on DBS for motor functional recovery in human and animal models. We highlight research providing insight into underlying cellular and molecular mechanisms. A literature review via Web of Science and PubMed databases from 1990 to May 29, 2024, reveals a growing body of evidence for therapeutic DBS in SCI recovery. Advances in techniques like optogenetics and whole-brain tractogram have helped elucidate DBS mechanisms. Neuronal targets sites for SCI functional recovery include the mesencephalic locomotor region (MLR), cuneiform nucleus (CNF), and nucleus raphe magnus (NRG), with pedunculopontine nucleus (PPN), periaqueductal gray (PAG), and nucleus ventroposterolateral thalami (VPL) for post-injury functional recovery treatment. Radiologically guided DBS optimization and combination therapy with classical rehabilitation have become an effective therapeutic method, though ongoing interventional trials are needed to enhance understanding and validate DBS efficacy in SCI. On the pre-clinical front, standardization of pre-clinical approaches are essential to enhance the quality of evidence on DBS safety and efficacy. Mapping brain targets and optimizing DBS protocols, aided by combined DBS and medical imaging, are critical endeavors. Overall, DBS holds promise for neurological and functional recovery after SCI, akin to other electrical stimulation approaches.

## Introduction

**Figure 1:**
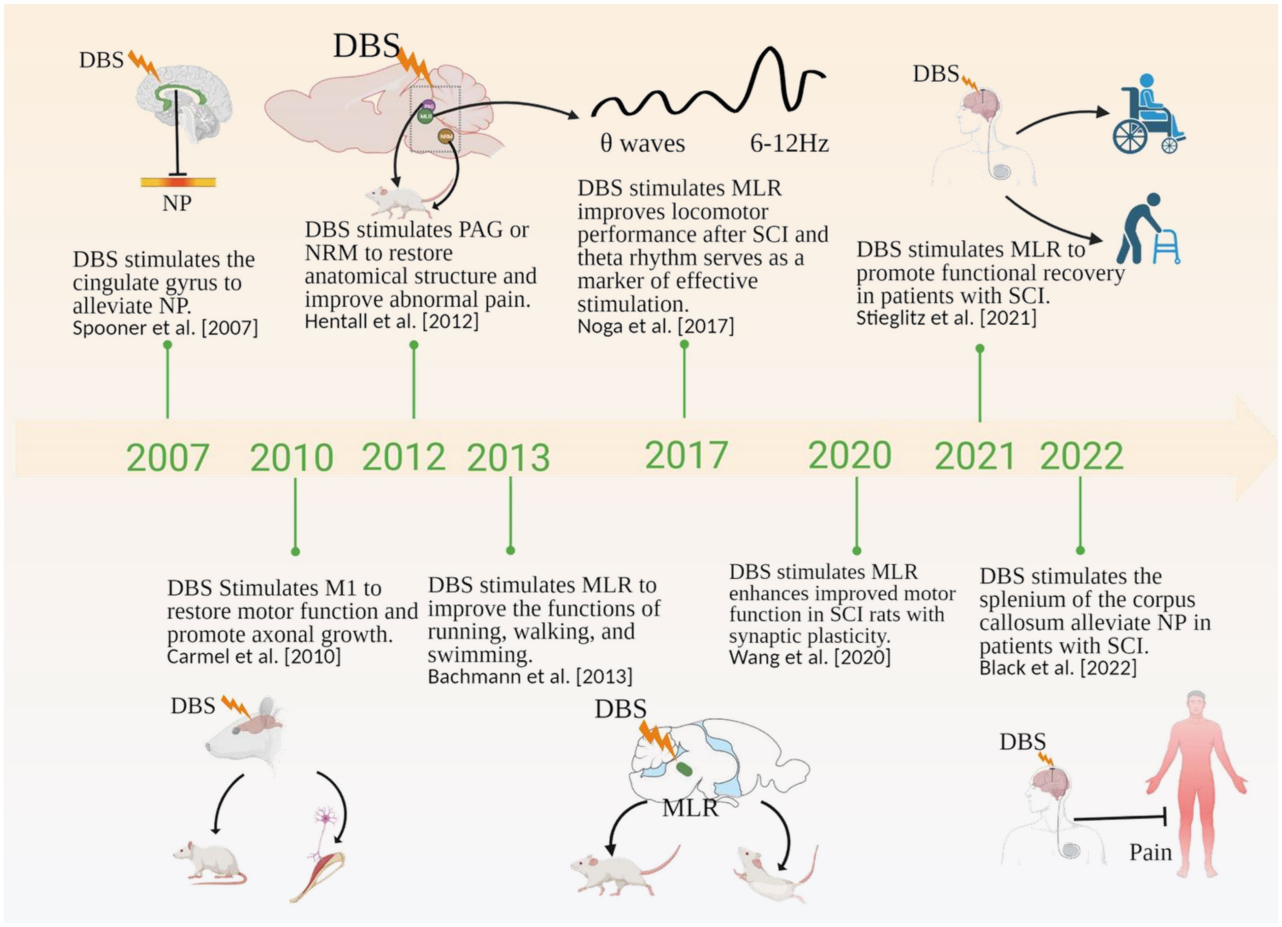
Timeline showing the role of DBS in SCI in the literature. NP, Neuropathic Pain; PAG, periaqueductal gray; NRM, nucleus raphe magnus; MLR, mesencephalic locomotor region. Created with BioRender.com.

The standard of care for spinal cord injury patients consists of acute surgical intervention and intensive post-acute rehabilitation. Despite the positive recovery of sensorimotor functions achieved by these methods, complete functional recovery is largely limited (1).There is very low evidence that these intervention improves ASIA motor score (AMS) in the short term (2), moreover, the anticipated undesirable effects include any major complication, surgical device-related complications, pressure ulcer, sepsis secondary to systemic infection, neurological deterioration, need for tracheostomy, and cardiopulmonary dysfunction (3). Therefore, finding a more effective and safe treatment plan is the top priority. The approach of electrical stimulation of the spinal cord to enhance functional recovery has yielded promising results. This review aims to provide an overview of the neural circuitry remodeling mechanisms of deep brain stimulation (DBS) after spinal cord injury, which may contribute to the improvement of motor function. DBS has been successfully used in the treatment of various movement disorders for years (4). The body of literature regarding DBS for SCI recovery has steadily increased in the past decade. In fact, DBS is currently also being applied post-SCI with promising results (5). However, the majority of research on DBS for SCI have focused on its efficiency on SCI-related neuropathic pain, while very few have attempted to ascertain the effects of DBS on motor functional recovery after SCI. In addition, some intrinsic mechanisms involved in the beneficial effects of DBS have been identified, including neuronal circuit remodeling, and potential alterations in intracellular signaling induced by DBS (6). Pre-clinical models have shed important light on mechanisms and best approaches, while researchers continue to synthesize and interpret outcomes from heterogeneous applications and populations. By far, radiologically-guided DBS has demonstrated the greatest improvement in patient outcomes, in addition to allowing the refinement of functional targets as the body of literature evolves (Figure 1).

## Search strategy

The articles used in this review of DBS for SCI treatment were retrieved by replicating the search terms of Vanegas et al. (7). A narrative review of the literature was performed using the keywords “DBS,” “Spinal Cord injury,” and “motor function,” via Web of Science, Google Scholar and PubMed databases. The search strategy and selection criteria utilized keywords/terms: deep brain stimulation (MeSH Terms), neural circuits (MeSH Terms), Spinal cord injuries (MeSH Terms), neuromodulation (MeSH Terms), neuroplasticity (MeSH Terms), motor function (MeSH Terms).

*The inclusion criteria:* Studies providing preclinical and clinical motor function results after DBS treatment in SCI models. For animal study, retrospective or prospective clinical studies were included. Papers had to be published in English between January 1990 to May 29, 2024.

*The exclusion criteria:* Reviews, meta-analyses, and those written in a non-English language, and studies concerning DBS treatment SCI related- pain. Other articles for whom the full text could not be retrieved were excluded.

**Figure 2:**
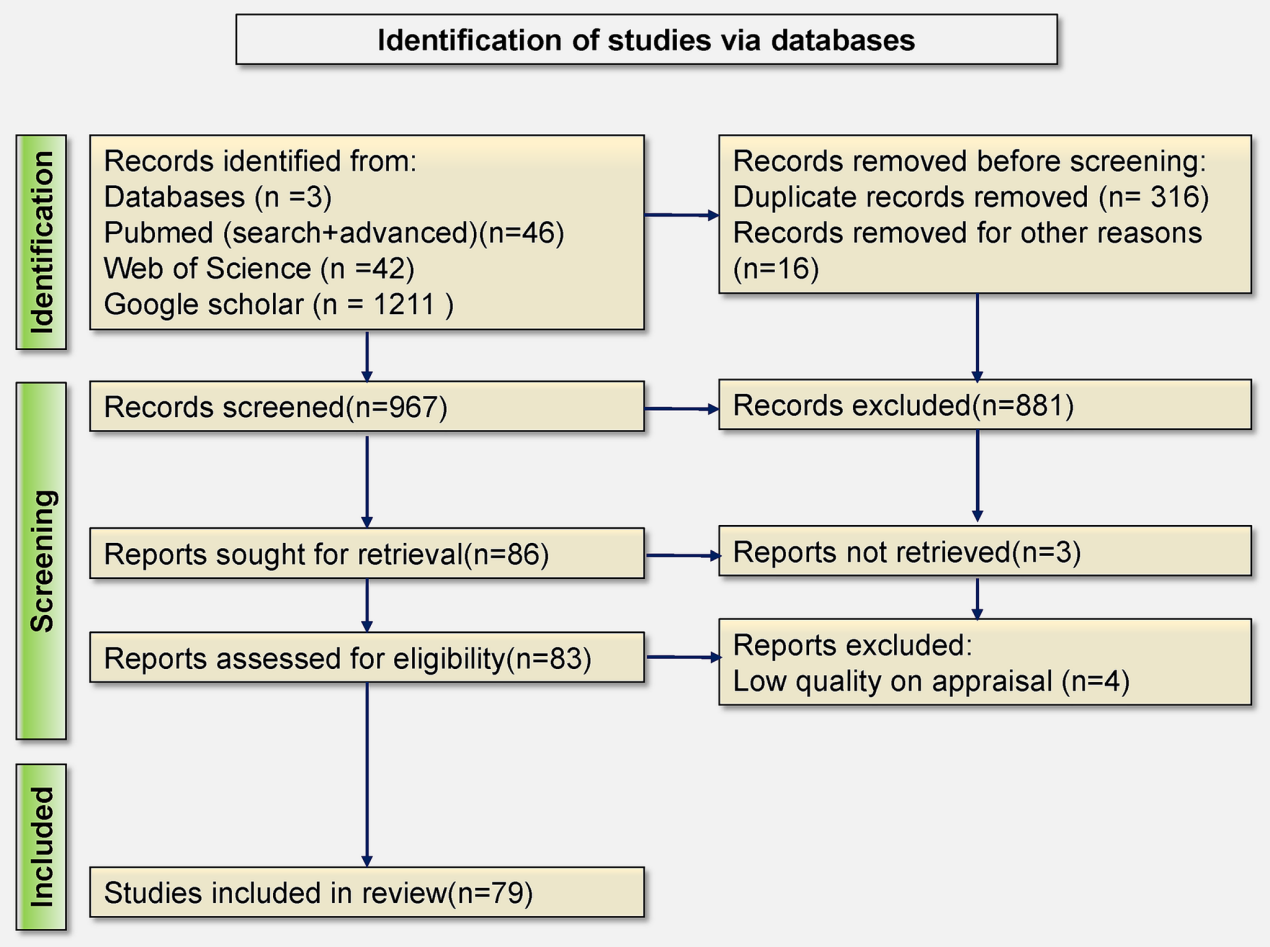
PRISMA flowchart depicting article selection.

*Selection Process:* The detailed study selection process is visually represented in Figure 2 within the PRISMA flowchart (8). Ultimately, we included 79 articles in our review. The protocol was registered with PROSPERO (registration ID: 598390).

*Quality assessment:* The assessment of the methodological quality of individual studies was conducted independently by two researchers (WyL and WY) according to a checklist designed by Van (9).

## Post-SCI neuronal circuit remodeling

After SCI, local circuits within the spinal cord may partially or completely lose their cortical spinal motor inputs. Complex corticospinal circuit remodeling ensues, demonstrating automaticity and spontaneous plasticity (10). While some spontaneous plasticity occurs to benefit the regeneration of the corticospinal tract, off-target re-wiring can be detrimental to recovery. Circuit remodeling facilitating functional recovery mainly operates via supraspinal axon re-growth to form compensatory circuits. Target selection of these axons is critical, with various competing relay neurons and axon guidance factors at play. Ultimately, evidence suggests this modulation is activity-dependent (11), and can thus be therapeutically utilized to improve motor function after SCI. Indeed, this has been the premise of neurorehabilitation and locomotor training for years (12, 13).

In the past decade, many strategies to facilitate neuromodulation after SCI have been developed, including stem cell implantation, epidural stimulation (ES) and DBS. In this review, we will focus on the body of evidence for DBS as a tool for neuromodulation, circuit remodeling, and functional recovery after SCI.

## Premise for neuromodulation via DBS in SCI

The study of high-frequency DBS for therapeutic purposes was pioneered decades ago. Since then, elegant hypotheses have been elaborated to explain the most complex aspect of DBS-its dynamic stimulatory properties (14). The synchronized parallel forebrain hypothesis (an extension of the rudimentary centrencephalic system initiated by Wilder Penfield) proposes that ablation results from high-frequency DBS when it targets synchronous neurons and stimulation when it targets asynchronous neurons. Since, the focus in DBS research has largely remained to target and activate residual neural pathways in such a way to activate locomotion (e.g., via central pattern generator networks), though use of DBS for stereotactic ablation has gained traction for the treatment of movement disorders (15). Recently, implantable electrical stimulation modalities have been increasingly used in combination with high-throughput computer simulators, which rapidly record circuit feedback to refine spatiotemporal selectivity and improve functional features (16).

The FDA approved DBS for use in Parkinson’s Disease (PD) patients in 2015 (17). Since, DBS has been trialed in human patients with largely positive outcomes (18, 19) observed from stimulation of the subthalamic nucleus (STN) and globus pallidus interna (GPi), including stable and longitudinal motor function improvement. More studies have since substantiated the efficacy of DBS as a surgical intervention for other tremor-based disorders (20, 21).

Deep brain stimulation typically consists of intracranial electrodes implanted surgically and connected to a subcutaneous impulse generator. The procedure has been generally well-tolerated (22). This finding was reported in a meta-analysis of randomized controlled PD trials, and may not translate to DBS for SCI patients. Despite any reservation, the use of DBS as a neuromodulatory therapeutic only continued to expand, recently gaining popularity as a therapeutic for diverse psychiatric disorders (23). Its efficacy in SCI patients appears to be variable and correlated with target region and stimulation parameters (24). As such, identifying and better understanding these targets and their role in recovery mechanisms is crucial for optimization of DBS. For example, the brainstem has become a therapeutic target of SCI due to its ability to coordinate locomotor systems via the integration of sensory, cognitive, endocrine, autonomic, and musculoskeletal systems in animal models (25). It additionally remains crucial to optimize techniques and stimulation parameters to achieve improved and sustained benefits while minimizing safety risks (26). Some advantages of DBS include its rather broad applicability to neurosystemic targets in addition to its dual hemispheric tolerability and customizability from the electrical source.

## Effects of DBS on motor function after SCI

**Figure 3:**
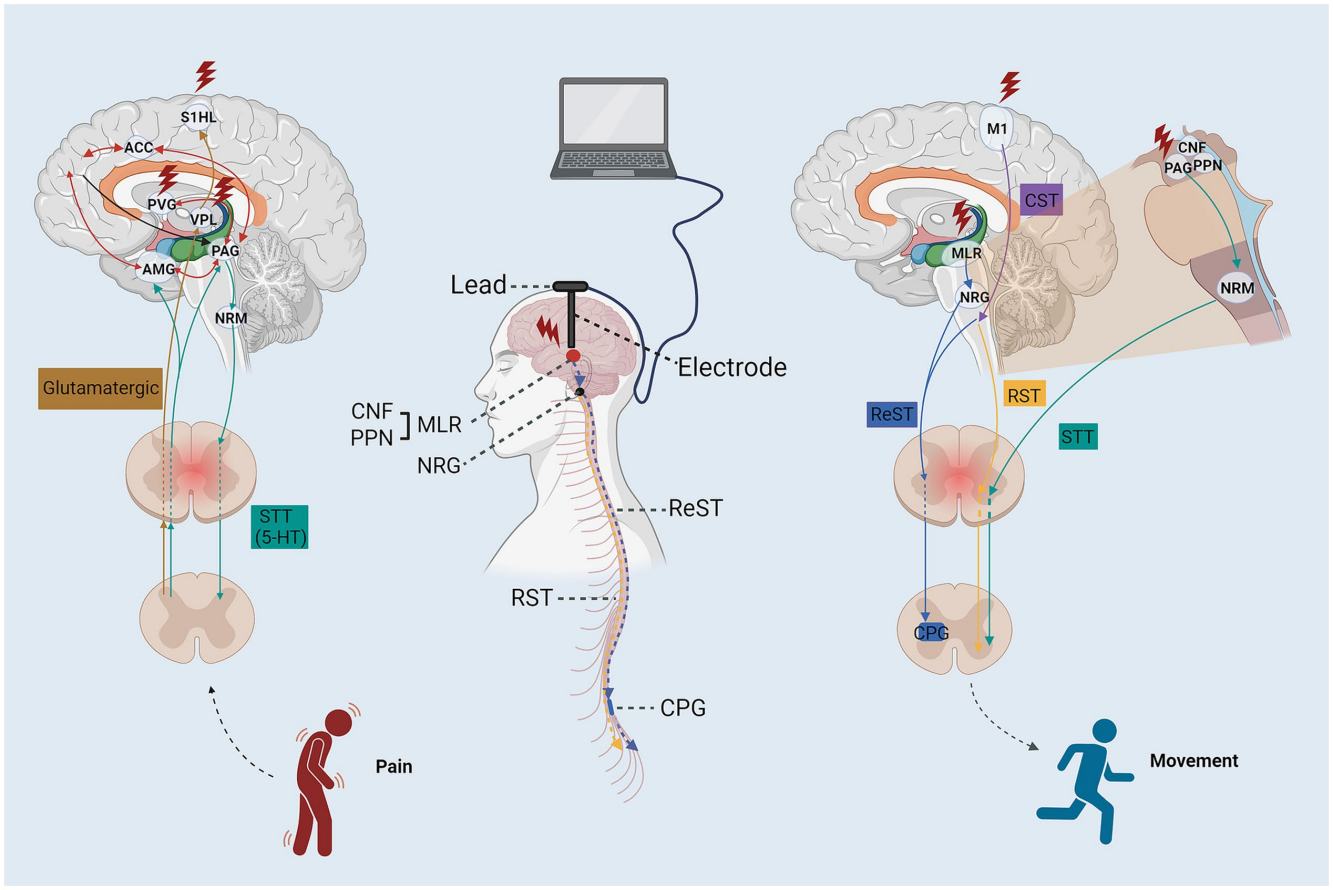
The schematic diagram of the stimulation targets and neural circuit for DBS enhance motor and sensory function after SCI. ACC, anterior cingulate cortex; ReST, reticulospinal tract; STT, spinothalamic tract; RST, rubrospinal tract; CTS, corticospinal tract; AMG, amygdaloid nucleus; PFC, prefrontal cortex; PVG, periventricular gray; S1HL, somatosensory cortex hindlimb cortex; VPL, nucleus ventroposterolateral thalami; PAG, periaqueductal gray; NRM, nucleus raphe magnus; PPN, pedunculopontine nucleus; NRG, gigantocellular reticular nucleus; CPG, central pattern generators.

Early animal studies of direct activation of the corticospinal tract through DBS of the internal capsule demonstrated increased axonal outgrowth of the CST in non-human primates (27). DBS selectively activates axons specific orientations by modifying the stimulation configuration, and selectively stimulating axons, substantially enhancing the potential clinical outcomes of DBS in SCI patients (28). Additional research has provided comparable insights into the effectiveness of DBS in rodent models of SCI subsequent to targeted activation of subcortical locomotor regions. Other studies have similarly shed light on the efficacy of DBS in enhancing motor function in rat SCI models following specific activation of subcortical locomotor areas (25, 29). Bachmann and colleagues demonstrated that an MLR stimulation paradigm was sufficient to recover locomotor strength in just 4 weeks, re-establishing near pre-lesion walking capacity of injured rats (25). Hentall and colleagues reported that stimulation of the raphe magnus or periaqueductal gray (PAG) in lesioned rats produced sustained improvements in locomotor performance and increased axon myelination and serotonergic terminals, noting that window of DBS treatment produced variable recovery (29). Other studies have reported functional improvements in hindlimb motor function (30) in response to stimulation, while others targeting neuromodulation of CnF observed accelerated forelimb locomotor recover and late-onset hindlimb activation and improved walking ability within 5 weeks (31). Several studies have revealed its application in improving motor function by targeting the subcortical motor area for stimulation in the animal SCI model (25). Differential functional recovery response across studies is likely due to degree of spared fibers within the injured spinal cord (variability of lesion protocol), target area stimulation, and DBS parameters (Figure 3).

To date, whether DBS could promote recovery of voluntary locomotor ability has not been clarified in humans. A current trial (NCT03053791) is underway for non-ambulatory SCI patients experiencing subchronic and chronic SCI. Based on experience gained from the first study participant in this clinical trial, the motor function of the SCI patients is most likely to benefit from MLR- DBS (5), which provides preliminary clinical evidence for the DBS in the restoration of motor functional recovery following SCI. The main endpoint for this human trial is enhanced locomotor recovery in chronic SCI patients undergoing intense neurorehabilitation (32). Nevertheless, few clinics evidence for the efficacy of DBS in the management motor functional following SCI, Instead, the vast majority evidence of SCI-related neuropathic pain patients who are the most likely to benefit from the DBS.

## DBS regulation of cortical circuits

The descending reticulospinal tract (RS) is prime conveyor or locomotor commands from the brain to intraneuronal circuits. After SCI, the number of spared fibers is typically insufficient for appropriate control of sublesion locomotor circuits (5). Some of the compromised locomotor circuits include central pattern generators (CPGs), responsible for alternating motion and syncing. There is some evidence that with training, electrical stimulation can re-activate these CPGs (33, 34). Importantly, there is evidence that lumbosacral CPGs can respond to external electric stimulation, even in the absence of sub-lesion response to supraspinal inputs (which has important implications for complete SCI patients) (35). Due to the input source of reticulospinal fibers, the MLR has become a major target of neuromodulation approaches in SCI treatment. A caveat of MLR-applied DBS for the treatment of SCI is the required residual reticulospinal fibers, which are only observed in incomplete SCI cases. Fortunately, the estimated rate of incomplete human SCIs outnumbers complete SCIs about 2:1 (36).

The rubrospinal tract (RuS) has been implicated in the recovery of cortical-dependent locomotion, including walking, climbing and swimming (37). DBS of the subcortical-cerebellar pathway demonstrated recruitment of the cortico-basal ganglia circuit during both resting state and voluntary movement (38).

By creating a whole-brain tractogram, the reconstruction of CST, pallidothalamic (PT), and cerebellothalamic (CBT) pathways are directly relevant to DBS activates particular axonal pathways (39, 40). Additionally, DBS can increase the functional connectivity of the motor and premotor cortex, enhancing motor coordination and response to mechanical stimulation.

**Figure 4:**
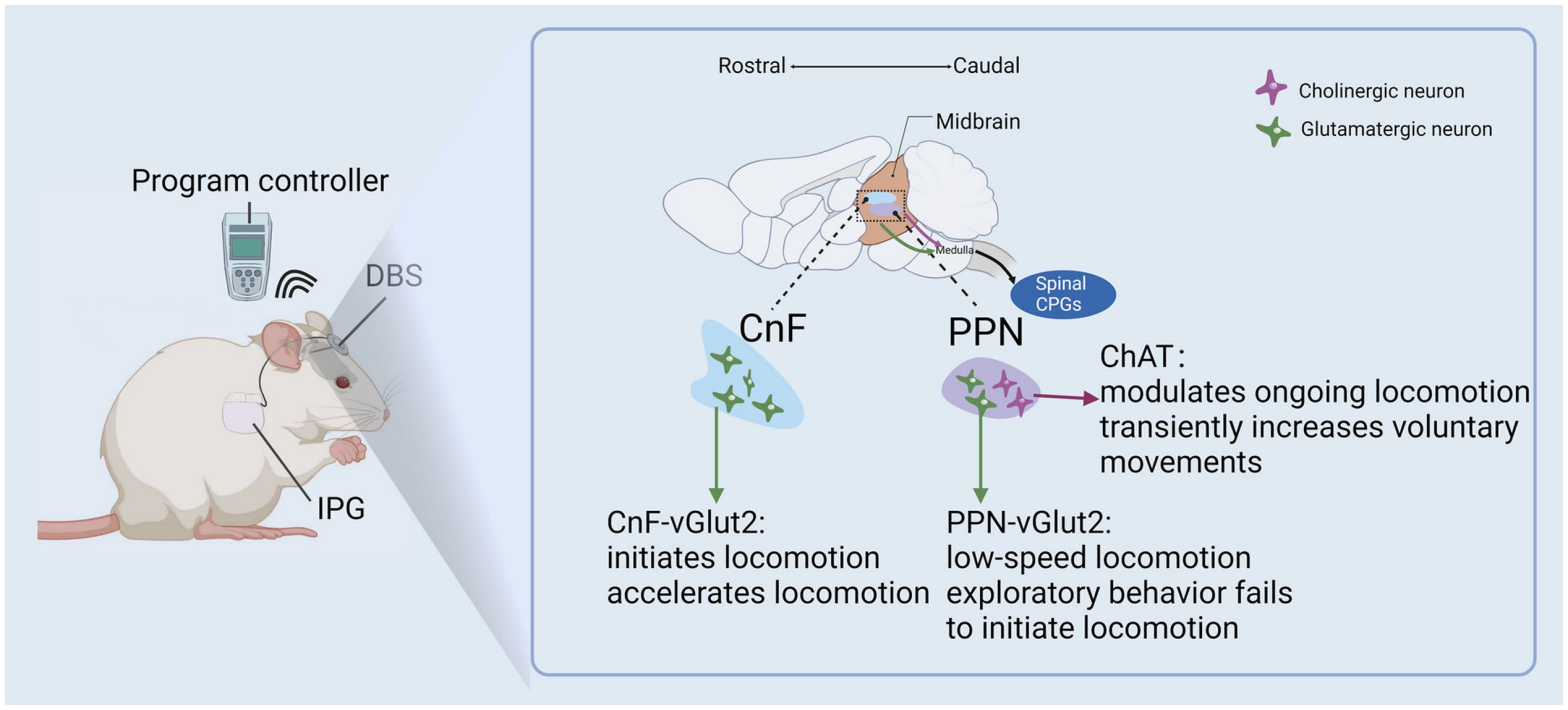
DBS of the MLR improve remodeling of cortical circuits after SCI.

Sophisticated studies have revealed that glutamatergic MLR neuron activation is sufficient to initiate and regulate locomotor acceleration (41, 42). Similar observations have been reported on the function of glutamatergic CnF neurons during the initiation and regulation of gait (43). Conversely, glutamatergic PPN neurons may play an inhibitory role in locomotion, regulating variable pre-motor properties such as motor tone (43). It has been proposed that one of the ways MLR stimulation may be rehabilitative to the injured spinal cord is by increasing synaptic plasticity among surviving reticulospinal pathways (44) (Figure 4). Indeed, many neuromodulatory approaches have been adapted to leverage the spontaneous compensatory sprouting of proximal corticospinal fibers to promote functional reorganization of spinal sensorimotor networks (45) in ways akin to the activity-dependent plasticity that assists in functional recovery during post-injury rehabilitation (46). Raphe magnus neuron stimulation via inputs from the more accessible periaqueductal gray (PAG) have been safely accessed for drug-refractory pain treatment by DBS in human (47). In a rat model of SCI, 4–7 day stimulation protocol produced motor recovery and myelination (29). However, to-date, the large majority of DBS studies targeting the PAG have focused on the treatment of post-SCI functional recovery (24). Overall, DBS can enhance corticospinal circuit remodelling and elicit complex, meaningful locomotor patterns by utilizing preserved complex spinal cord circuits following SCI (43). SCI patients with preserved spinal cord circuit fibers may have a beneficial outcome from DBS treatment, which may potentially enhance their daily functioning.

Other circuits to be investigated more deeply include those that negatively affect functional recovery after SCI. For example, long ascending propriospinal neurons that regulate central pattern generator (CPG) to contribute to left–right coordination of hindlimbs have been shown to negatively impact the recovery of hindlimb locomotor function in a rat SCI model (48). Recently, the idea of silent synapse (AMPAR-deficient, glutamatergic post-synapse contacts) remodeling after SCI has also been proposed, prompting new questions about how neuromodulatory approaches may be applied to normalize these in the corticospinal motor circuitry post injury (45). Many SCI patients experience motor spasticity that negatively impacts quality of life. While no DBS studies have investigated its effect on post-SCI spasticity, recent meta-analysis and systematic review has outlined that repetitive transcranial magnetic stimulation (rTMS) significantly reduced spasticity in a population of multiple sclerosis patients (49). More research on the neuromodulation of targets responsible for voluntary muscle contraction (e.g., global pallidus internus) is needed in the SCI population.

## Cellular and molecular mechanisms of DBS neuromodulation

Toward an improved understanding of the molecular mechanisms conferring the therapeutic and neuroadaptive effects of deep brain stimulation, one study has reported that DBS-derived synaptic plasticity is mediated in part by elevations in brain derived neurotrophic factor (BDNF) and downstream synaptic proteins (30). Other models of electrical stimulation have similarly observed upregulated BDNF (50, 51) DBS also elevates the BDNF receptor tropomyosin-related kinase B (TrkB), p70 ribosomal S6 protein kinase, and protein kinase B (30). This pathway is corroborated by other electrical stimulation paradigms including peripheral nerve stimulation (52). The BDNF/TrkB pathway is thought to activate neuroprotective, neuroplasticity, and pro-regenerative signals to aid in functional remodeling after SCI (53).

Improved understanding of the anatomical contributors to locomotor initiation and control come from optogenetic studies revealing the distinct physiological and functional subpopulations. For example, the glutamatergic CnF population were found to initiate short latency locomotion (41, 43) while ventrally-adjacent glutamatergic PPN neurons are thought to either not contribute to locomotor initiation or exploratory locomotion only (41). A study of the MLR in freely moving micropigs has provided electrical characterization including off-target effects of brainstem cardiovascular centers (54). This study corroborated the role of DBS on locomotor initiation and frequency-dependent speed regulation as well as (54). There are no clear boundaries for the sub-regions of the MLR, particularly in higher-order vertebrates. The PPN is largely characterized by its neuronal subpopulations, including cholinergic, glutamatergic, and GABAergic neurons. While optogenetic studies have proposed cholinergic PPN neurons play a role in locomotion (41), other studies have reported little to no effect on locomotion or speed (55).

**Figure 5:**
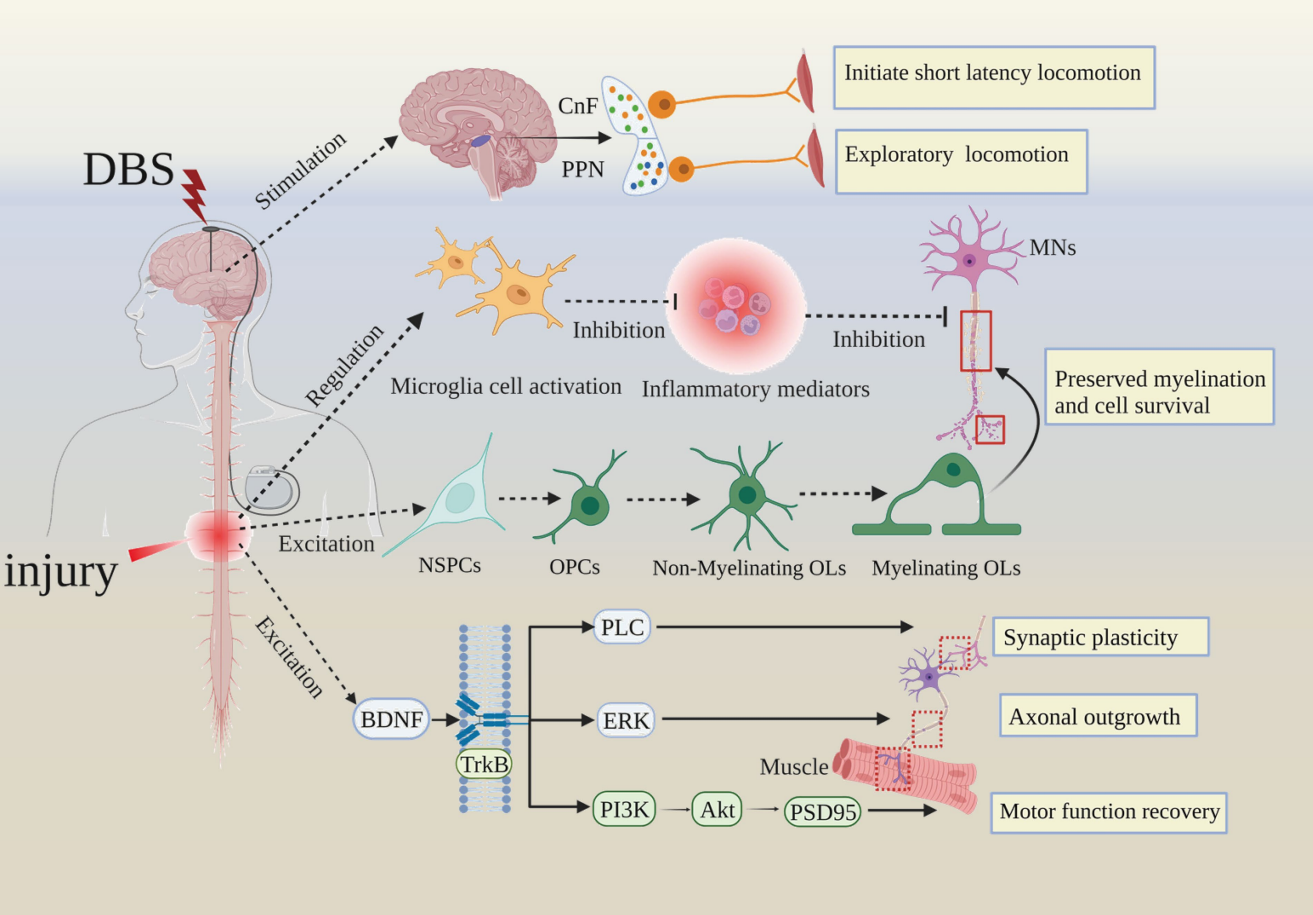
Summarizing the cellular and molecular mechanisms of DBS improves SCI prognosis. Akt, Protein Kinase B; CnF, cuneiform nucleus; MNs, motor neurons; NSPCs, neural stem/progenitor cells; OPCs, Oligomeric proanthocyanidins; Ols, oligodendrocytes; PLC, phospholipase C; ERK, extracellular regulated protein kinases; PI3K, Phosphoinositide-3 kinase; PSD95, postsynaptic density-95.

Evidence from electrical stimulation studies has shown that while functional improvement is achieved with external stimulation after SCI, long-lasting functional improvements can be observed chronically, in the absence of the external signal (37, 56). This indicates that sustained neuroplasticity occurs after therapeutic intervention to support long-term recovery. Indeed, epidural electrical stimulation (EES) paradigms have demonstrated robust and specific transcriptional and neurotransmitter changes to subsets of specialized interneurons in response to EES (57). In reality, the mechanisms of DBS on SCI remodeling are likely diverse and cumulative, including proximal and circuit-wide electrical and chemical effects to modulate activity, plasticity, and anatomical re-organization over time (58). As technical advances allow for more sophisticated experiments, we expect to increasingly delineate the contributory mechanisms and functional circuits that make-up the pathogenesis of SCI (Figure 5).

## Trajectory and protocol optimization of DBS for SCI

One of the remaining challenges of DBS for neuromodulation of SCI is identifying precise and effective brain regions to achieve maximal therapeutic efficacy and minimizing off-target effects. This type of trajectory planning is required in part due to the poor characterization of many regions of the brain related to locomotor regulation in humans (59). Mapping anatomical substrates with their maximal therapeutic response can help build predictive tools for clinical decision-making and is a step toward fully personalized application of DBS.

One such approach is probabilistic stimulation maps (PSM) derived from retrospective DBS datasets. These maps are based on activation volumes observed by medical imaging across heterogenous populations. In short, using MRI imaging and activation volume modelling, PSMs were created and described in terms of their interactions with surrounding anatomical structures, defining areas of above-mean and below-mean response for each patient cohort. However, the major of limitations of current approaches to PSMs is the accuracy of predefined DBS targets. PSMs predictive capability was not high and likely reflects both technical limitations of the mapping technique as well as the merging of numerous patient data on MRI models, which failed to consider each patient’s individual neuroanatomical location and variations after SCI. Consequently, PSMs alone are currently insufficient as a robust and consistent predictor of clinical outcome (60). One center collected 15 years’ worth of data from 482 patients, noting high correlations between PSMs and actual patient activation volumes (61). Another group created a machine learning model to predict optimal vs. non-optimal DBS parameters in a prospective cohort of 67 PD patients who underwent fMRI-observed stimulation. The predictive response maps generated were 88% accurate and maintained their topographic patterns across subtherapeutic and supratherapeutic stimulation parameters (62). More mapping efforts are reasonably expected. One important consideration as these trajectory planning strategies move forth are the different analytical methods being utilized, as these have substantial variability even within the same datasets (60). Although PSM accuracy needs to be thoroughly examined, it may enhance the comprehension of the effects of DBS and have potential applications for DBS targets in the treatment of SCI.

Electrophysiological mapping of MLR has been most notably investigated for the treatment of movement disorders with DBS. Some investigations have used local field potential monitoring across subregions of the MLR to examine the effectiveness of these targets in animal models. For example, voluntary locomotion in normal rats produces synchronized theta oscillations (6–12 Hz) in the MLR in intact rats and other regulatory regions of locomotor control. Interestingly, these theta oscillations persist after SCI in the MLR and may be useful in target planning during therapeutic DBS (63). On the other hand, the optogenetic stimulation of the PPN subregion at ~40 Hz has been shown to reliably induce locomotion in animals (64). One study used a combination of electromyographic recording, genetic manipulation, and anatomical analysis in an animal SCI model, reporting that glutamatergic neurons within the CnF improve motor performance in the hindlimb muscles while glutamatergic PPN neurons inhibit locomotion when activated (65). Those findings are consistent with previous reports of DBS of the CnF, which produced enhanced motor drive in rats with incomplete SCI, allowing high-intensity locomotor training after injury (44). Finally, a micropig model of radiologically-guided DBS found that functional stimulation of the MLR was based on deliberate targeting of a PPN cholinergic population and a CnF cluster dorsal to that (54). Thus, as more detailed evidence becomes available, investigators must work toward an increasingly defined “map” of the MLR for DBS targeting.

Finally, it remains important to optimize lead implantation and stimulation protocols to maximize efficacy and minimize risks of adverse events. Stereotaxic microelectrode implantation is an effective surgical approach for the treatment of movement disorders. Stereotaxic head frames are used to increase accuracy of electrode placement and trajectory, and coupled with peri-operative imaging, can be very accurate. Advances in accessibility of 3D printing, robotics, and real time neuroimaging are further improving precision in surgical implantation (66–68).

Parameter optimization is equally important to resolve in the field of therapeutic DBS, and likely accounts for as much variability as anatomical factors. Several animal models of SCI have demonstrated maximal therapeutic responsiveness to low-threshold stimulation for voluntary locomotion (25, 63, 69). Other studies report that step-wise increases in DBS stimulation frequency of the MLR not only initiate locomotion but increase locomotor frequency (54). There are distinctions between DBS targets CnF and PPN even if their mechanisms are similar. By comparison we found that CnF may be more important than PPN in the initiation and regulation of gait, and facilitating speed of locomotion based on animals’ research (43, 70).

## Model and stimulation parameters

Other pressing objectives in the field of DBS remains protocol standardization for improved summary of research findings and downstream applications. For example, one review synthesized DBS for Alzheimer’s disease, analyzing targets, stimulation frequency, duration, intensity, and treatment time from disease onset (71). While homogeneity of disease may limit such study or protocol synchronicity in humans, animal models for research are an opportunity for consolidation of knowledge generated from a standardized approach, and may thus allow for testing and validation of optimal DBS parameters for human extrapolation. In conjunction with advanced techniques such as MRI and optogenetics for trajectory mapping, refining stimulation parameters may maximize the therapeutic effect of DBS in SCI patients.

Other DBS protocol considerations include the orientation of the electrode relative to the target (28), signal intensity (pulse, frequency and amplitude), and stimulation mode (monopolar, bipolar, tripolar, etc.). For example, in animal models of severe SCI, gait improvements were only achieved with equivalent stimulation current as required to initiate involuntary movement in healthy controls (72). In humans, where device longevity is a concern, bipolar stimulation appears to improve the life of the mean pulse generator compared to monopolar mode (73). DBS parameters not only play an important role in its therapeutic efficacy, they may be as crucial for safety and to prevent off-target adverse events as patient characteristics (surgical and pre-surgical) (74).

Finally, combining DBS with other interventions may enhance the efficacy of DBS as a monotherapy, as has been the case with other electrical neuromodulation approaches. For example, transcutaneous electrical nerve stimulation in combination with functional task practice promotes corticomotor excitability in patients with chronic cervical SCI, though stability of the response was not examined beyond 30 min (75).

In addition to isolated neruomodulatory approaches, combined strategies are theorized to yield significant improvements due to the overlap of spinal locomotor neurons activated during treatment (e.g., DBS/spinal cord stimulation) (44). Indeed, a variety of combinatorial techniques have demonstrated neuroplasticity-driven functional recovery after SCI particularly in corticospinal circuits (31, 54, 76), including combined electrostimulation and pharmacologic approaches (72, 77).

Meanwhile, the demand for pairing radiological imaging with DBS patients continues to grow in order to accommodate the need for personalized evaluation, however the risks aversion of such approaches remain high due to stringent contraindications (78). Recent studies have challenged these manufacturer-based guidelines, providing safety data on 102 patients with no adverse events or DBS impedance, and only a 1.4% intracranial artifact around the implant (79). The initial clinical report on the effects of DBS in patients found that stimulation frequencies >130 Hz were optimal for inducing locomotor movements (80). However, in various animal models suggesting 40–60 Hz with broad pulse sizes (200–1,000 μs) was the most efficient range to elicit locomotion (54). Continuing efforts to expand the safe use of radiological imaging include standardization across centers, device specifications, radiofrequency exposure characteristics, magnetic field strengths, and patient positioning protocols. As referred to previously, increased monitoring during DBS is likely to refine and optimize therapeutic efficacy (see Table 1).

**Table 1:** The preclinical and clinical study of DBS in SCI.

Study	Study details	Brain target	Physiological changes	Functional changes	Stimulation parameters	Analysis methods for mechanisms
Carmel et al. (81)	Preclinical study: female rat (225–275 g; n = 10)	Unilaterally-M1 [B:2 mm /3.5 mm]	DBS induced CST axon terminations outgrowth in the ipsilateral spinal cord	DBS improve skilled motor performance following a unilateral SCI	333 Hz,0.2 ms duration, 6 h daily for 10 d	Behavioral testing; neural tracer technology; stereological analysis; regional axon length analysis
Hentall et al. (29)	Preclinical study: female rat (250–275 g; n = 64) T8 SCI	Midline-NRM [caudal: 2.3 mm, ventral: 0 mm] R-PAG [ML: 0.7 mm, rostral: 1.2 mm, above the interaural line:4 mm]	DBS (NRM or PAG) promoted the recovery of myelinated axons in perilesional white matter	DBS improve sensory, motor and anatomical recovery following incomplete SCI	8 Hz,1 ms,30 μA 12 h daily over 4–7 days	Behavioral testing; histological analysis
Bachmann et al. (25)	Preclinical study: female rat (220–250 g) T10 SCI	Unilaterally-MLR [B: −7.80 mm, L: +2.00 mm, D: −5.80 mm/0°]	DBS (MLR) activated the supraspinal motor control pathway from the medial brainstem to the lumbar spinal cord	DBS improved the residual locomotor performance after severe SCI	25,50,75 and 100 Hz,0.5 ms Awake animals: 50 Hz, 0.5 ms	Behavioral testing; neural tracer technology; EMG recordings
Noga et al. (44)	Preclinical study: female rat (240–350 g; n = 28) T9 SCI	L-MLR [AP:0.7–1.2 mm, ML:2.0 mm, DV:6.2 mm]	DBS (MLR) promoted the presence of theta rhythms in LFPs	DBS improved the locomotion after SCI.	10–70 Hz (10, 20, 50 and 70 Hz),0.2, 0.5, 1.0 and 2.0 ms	Behavioral testing; EMG recordings; immunohistochemistry
Wang et al. (30)	Preclinical study: male rat (250–300 g; n = 36) T10 SCI.	B-MLR [AP: −7.8 mm, ML: +2.0 mm, DV: −5.0 mm]	DBS improves synaptic plasticity by targeting BDNF, and mTOR	DBS improved hindlimb motor function in SCI rats	100 Hz, 0.5 ms half an hour per day for4 weeks.	Behavioral testing; western blot
Bonizzato et al. (72)	Preclinical study: female rat (200–220 g) T8 SCI.	L-PPN [AP: −7.9 mm ± 0.05, DV: −6.5 mm, ML: 2 mm]	DBS promotes the reconstruction of the remaining motor circuit	DBS promoted the SCI rats volitional walking	40 Hz, 200 μs, 50–250 μA 5 days/week 30 min /day	Immunohistochemistry; neuromorphological evaluation; electrophysiology
Hofer et al. (31)	Preclinical study: female rat (220–250 g; n = 127) T10 SCI	L-CNF [AP: −7.8 mm, DV: −5.1 to −5.5 mm, ML: +2.0 mm]	DBS activated spared descending brainstem fibres	DBS improves motor recovery in the subchronic and chronic SCI phases.	50 Hz, 0.5 ms	Behavioral testing; histological analysis; neural tracer technology
Spooner et al. (82)	Clinical study: male patients (40 year; n = 1) C4 level spinal cord injury	R-PVG [AP: −8.2 mm, lateral: +4.2 mm, vertical: +1.1 mm] B-Cingula [20 mm]	The analgesic effect of DBS on bilateral cingulate gyrus is superior to that of PVG stimulation	DBS improves functional recovery in a complete spinal cord injury	PVG: 20 Hz Cingula: 130 Hz 1-week blinded stimulation trial prior	VAS evaluation

PVG, periventricular gray matter; CNF, cuneiform nucleus; PVG, periventricular gray matter; MLR, mesencephalic locomotor region; PPN, pedunculopontine nucleus; VAS, visual analog scale; B, bilateral; R, right; AP, antero-posterior; L, left; NS, no stimulation; ML, mediolateral; DV, dorsoventral; B, bregma; L, lambda; D, dura; BDNF, brain-derived neurotrophic factor, mTOR, the mammalian target of rapamycin; LFPs, local field potentials.

## Summary

Given that the quality of evidence for DBS for spinal cord injury in humans is very low, and that effective brain targets in animal models are uncertain, recommendations for the use of DBS in SCI patients remain uncertain. To determine the precise effects of DBS-mediated neuroplasticity on functional recovery following spinal cord injury, large-scale clinical trials and studies utilizing large animal models are required. Currently, there are very few clinical reports on DBS-related motor recovery in SCI patients available in databases, the first human clinical trial is underway to assess the impact of DBS on SCI populations (NCT03053791). Based on the literature a proposal for the ideal DBS treatment in SCI candidates may be an individual with motor incomplete SCI (confirmed by clinical and MRI examinations) and preservation of sacral function. In addition, medical imaging of DBS is a major problem, and radiological guidance may be needed to place, evaluate, and reconfigure DBS, particularly in light of more recent developments and intricate directional electrodes. The proposed DBS protocol is predicated on pre-clinical studies which target the MLR/DBS with low frequency (≤50 Hz) at medium to broad pulse widths. Optimal stimulation parameters will have to be determined for each patient individually as reference values from human patients are not yet available. Moreover, a strategy to dissect and comprehend the distinct neuronal subpopulations and their exact location for DBS treatment in SCI patients is required, which will clarify the DBS’s neuronal targets, in which the MLR has gained scientific and clinical interest as target for DBS to improve motor recovery after SCI with the CNF being proposed as the primary therapeutic target in recent rodent studies. A particular challenge for preclinical translation to human clinical research remains DBS targets accuracy. While the PPN/CNF and their microstructure of rodents are currently well-characterized, the human PPN/CNF is inadequately described (41). Therefore, a more comprehensive description of the macroanatomy and microanatomy of the human MLR is urgently required. Finally, there is a significant trend in combination therapy models, such as the application of DBS during post-injury exercise training, safe pharmacological cocktails or stem cell implantation. The literature reviewed suggest that MLR-DBS combined with rehabilitation methods or EES, including gait rehabilitation or intensive locomotor training, might facilitate motor recovery after SCI. In conclusion, sustained characterization of neuroplasticity after SCI and the development of modulated approaches such as DBS are expected to promote neurological/functional recovery in SCI patients. Certainly, advances in the field of DBS and other methods of electrical neuromodulation have revolutionized the long-held belief that SCI is irreversible.
